# Forests and ozone: productivity, carbon storage, and feedbacks

**DOI:** 10.1038/srep22133

**Published:** 2016-02-22

**Authors:** Bin Wang, Herman H. Shugart, Jacquelyn K. Shuman, Manuel T. Lerdau

**Affiliations:** 1 Department of Environmental Sciences, University of Virginia, PO Box 400123, Clark Hall, 291 McCormick Road, Charlottesville, VA 22904-4123, USA

## Abstract

Tropospheric ozone is a serious air-pollutant, with large impacts on plant function. This study demonstrates that tropospheric ozone, although it damages plant metabolism, does not necessarily reduce ecosystem processes such as productivity or carbon sequestration because of diversity change and compensatory processes at the community scale ameliorate negative impacts at the individual level. This study assesses the impact of ozone on forest composition and ecosystem dynamics with an individual-based gap model that includes basic physiology as well as species-specific metabolic properties. Elevated tropospheric ozone leads to no reduction of forest productivity and carbon stock and to increased isoprene emissions, which result from enhanced dominance by isoprene-emitting species (which tolerate ozone stress better than non-emitters). This study suggests that tropospheric ozone may not diminish forest carbon sequestration capacity. This study also suggests that, because of the often positive relationship between isoprene emission and ozone formation, there is a positive feedback loop between forest communities and ozone, which further aggravates ozone pollution.

Interactions between forests and the atmospheric pollutants are a crucial component of Earth System Science, but the impacts of changes in tree-species composition on ecosystems and the atmosphere are not yet well understood. Most long-term efforts to examine pollutant interactions with forests have relied on models based on process-level studies at biochemical and physiological scales^1^,^2^,^3^. These models do not explicitly consider variability among species, notably the impacts that growth and competition among species can affect system-level metabolism. Using an individual-based ecosystem model, we examined how species-specific variability in responses to the most important atmospheric pollutant in North America, ozone (O_3_)^2^, interacts with these higher-order processes and modifies functions at the community, ecosystem, and biogeochemical scales.

At cellular-to-organ scales, the impacts of O_3_ on plants are relatively well understood — ozone causes cellular damage; induces reduced stomatal conductance; eventually decreases carbon dioxide (CO_2_) assimilation rates and produces visible leaf injury^4^,^5^,^6^. These effects often accelerate senescence, diminish leaf area and biomass, and reduce productivity^4^,^5^,^7^,^8^. These responses promote the inference that O_3_ pollution should reduce forest ecosystem productivity and suppress terrestrial carbon sequestration^1^,^2^,^3^.

This inference ignores the differences among tree species in their sensitivity to O_3_^4^,^8^. These differences in sensitivities potentially mediate competitive interactions, giving O_3_-tolerant species that are competitively inferior in low-O_3_ environments advantages in high-O_3_ situations^5^,^9^,^10^,^11^. Understanding this complex problem requires consideration of both the diversity of species and sizes of trees in a forest, including their metabolic properties and competitive interactions. Such insights are particularly difficult to obtain in forests because of the long generational times that are associated with trees. Some studies have tried to conduct ecosystem-scale forest O_3_ experiments in the context of free-air carbon enrichment (FACE) experiments, but logistical limitations have required these studies to focus on a limited set of species and for a relatively short time period^12^,^13^,^14^.

The forest response to ozone is a complex mixture of the responses of individual trees of different species and sizes. The homogenization of this complexity can be lost in the aggregation necessary to construct ordinary-differential-equation-based process-models of ecosystem dynamics. An approach to overcome this difficulty is to simulate each of the trees in a forest ecosystem using individual-based models (IBMs)^15^,^16^. Here, we use a class of IBMs known as gap models to study the complex relationships among species-level variability in growth, ozone sensitivity, and ecosystem processes.

Gap models are IBMs that simulate growth, mortality, and regeneration of all individual trees in a ~0.10 *ha* plot in a forest, as well as their competition for light and other resources^15^. Such models have a rich history in community ecology^17^. Recent advances in computational power have allowed current versions of these models to explicitly simulate compositional and structural dynamics and to link these dynamics to ecosystem and biogeochemical processes. This study uses UVAFME^18^ (University of Virginia Forest Model Enhanced, Supplementary Fig. 1) to simulate the successional dynamics of species composition and structural change of a typical temperate deciduous forest in the southeastern USA, a region that is well studied in terms of forest succession and whose component species have been characterized with respect to their O_3_ sensitivity and competitive relations^19^.

## Results

**Figure 1:**
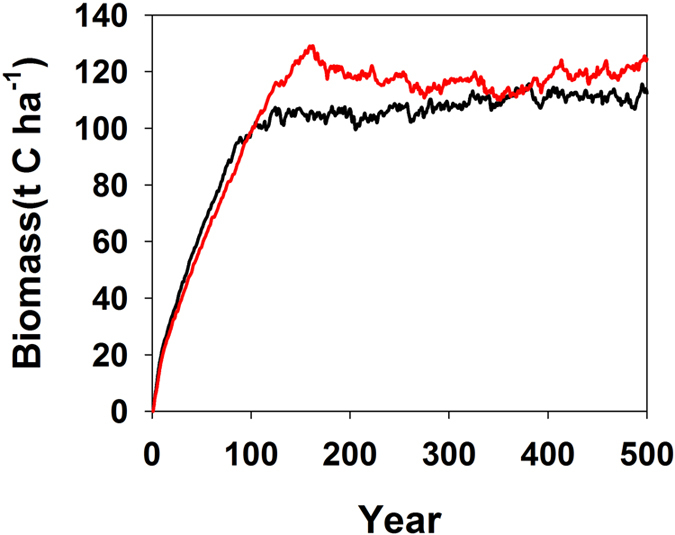
Successional changes in biomass carbon due to O_3_. Simulated biomass carbon response to O_3_ over 500 years succession. Dark and red line denote without and with O_3_ stress, respectively.

The simulated successional dynamics of this temperate deciduous forest over 500 years involve changes in 10 abundant species and 22 other species (Supplementary Fig. 2). Initially for a forest succession from an open plot, the ‘other’ species category, mostly composed of pioneer species, dominates the forest with approximately 50% of the total biomass (Fig. 1). Soon, both *Acer rubrum* and *Liriodendron tulipifera* become increasingly important, but *A. rubrum* eventually loses to the larger, faster-growing *L. tulipifera* trees, which persist and become dominant. After *L. tulipifera* declines over time, trees of four shade-tolerant oak species (*Quercus alba, Q. velutina, Q. rubra,* and *Q. prinus*) become increasingly important, together accounting for approximately 75% of the stand biomass at year 500. The composition of the forest stabilizes and is eventually dominated by the aforementioned oaks, along with *L. tulipifera*, and two maples (*A. rubrum* and *A. saccharum*). Correspondingly, total biomass becomes relatively stable starting around year 100 (Fig. 1). The simulated successional change resembles expected forest composition change in the southeastern USA^15^.

**Figure 2:**
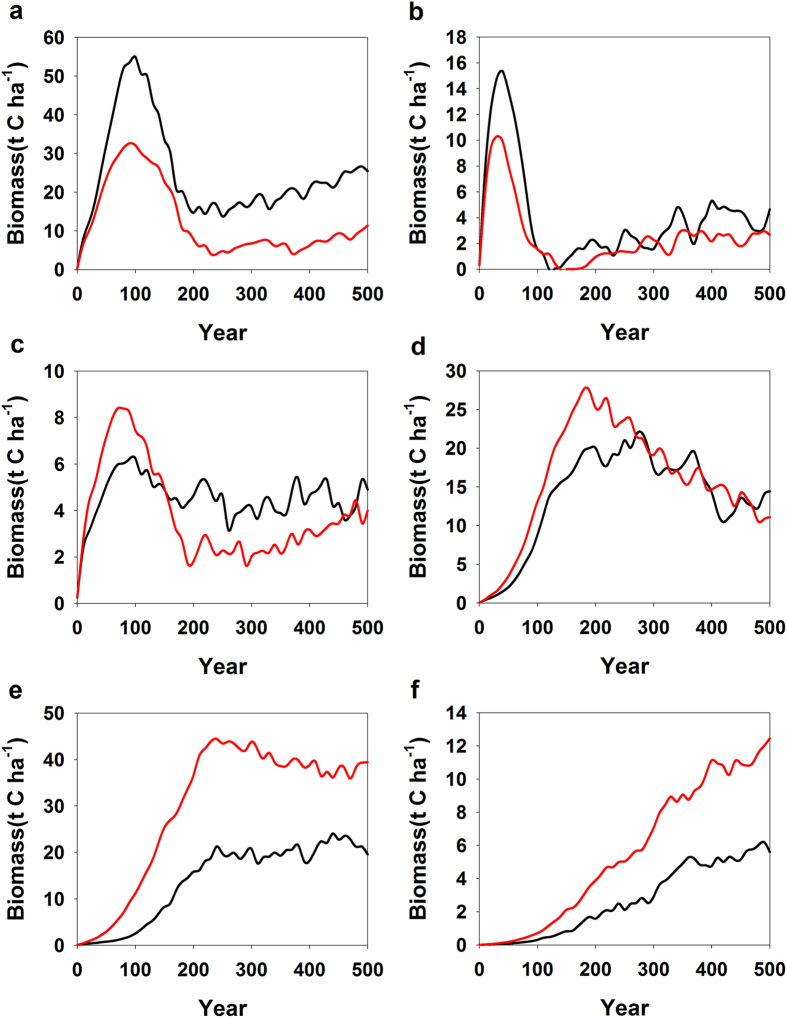
Successional responses to O_3_ by individual species within the simulated forest. Sensitive species: *Liriodendron tulipifera* (**a**) and *Acer rubrum* (**b**). Intermediate species: *Acer saccharum* (**c**) and *Quercus velutina* (**d**). Tolerant species: *Quercus alba* (**e**) and *Fagus grandifolia* (**f**). Dark and red line denote without and with O_3_ stress, respectively.

When O_3_ impacts on growth and competitive ability are included, the compositional changes differ from the case when O_3_ impacts are absent (Fig. 2). Generally, O_3_-sensitive species have lower biomass when exposed to O_3_ stress over succession (*e.g*., *L. tulipifera* and *A. rubrum*), but *A. rubrum* has almost same biomass at year 100 as the control case (Fig. 1). For species with an intermediate O_3_ sensitivity (*e.g., A. saccharum* and *Q. velutina*), biomass can be enhanced rather than diminished early in the stand dynamics. For resistant species (*e.g., Q. alba*), biomass is significantly enhanced by O_3_. An individual’s response to O_3_ is not absolutely determined by its intrinsic O_3_ sensitivity, and it can be modified through interactions with other species within the community (*e.g.,* ref. 9).

The differential sensitivity to O_3_ and release from competitive suppression result in a compensatory response from O_3_-tolerant species, with the result that forest biomass does not decline over time under high O_3_ conditions, although it is lower initially (Fig. 1). Forest carbon storage is also not suppressed by O_3_, and it gradually increases over time because of the unsuppressed net ecosystem productivity (Supplementary Fig. 3). These results differ from the logical inference emerging from coupled climate-biogeochemical cycling models (*e.g.,* refs 2,3) that do not include the species-specific individual-based metabolism and competitive interactions.

An important source of metabolic variation with respect to O_3_ in forests is the occurrence of isoprene-emitting taxa. Isoprene from forest trees dominates the annual global volatile organic compounds (VOCs) flux into the atmosphere^20^,^21^. Isoprene contributes to tropospheric O_3_ formation and aggravates O_3_ pollution under conditions of moderate to high nitrogen oxides^22^. Not all tree species, however, emit isoprene. About one third of tree species produce isoprene in both the eastern USA and tropical forests; low diversity boreal forests also consist of emitters (*e.g.,* spruce and aspen) and non-emitters (*e.g.,* pine)^23^. There are 10 isoprene-emitting species identified in this simulated forest (Supplementary Table 1).

**Figure 3:**
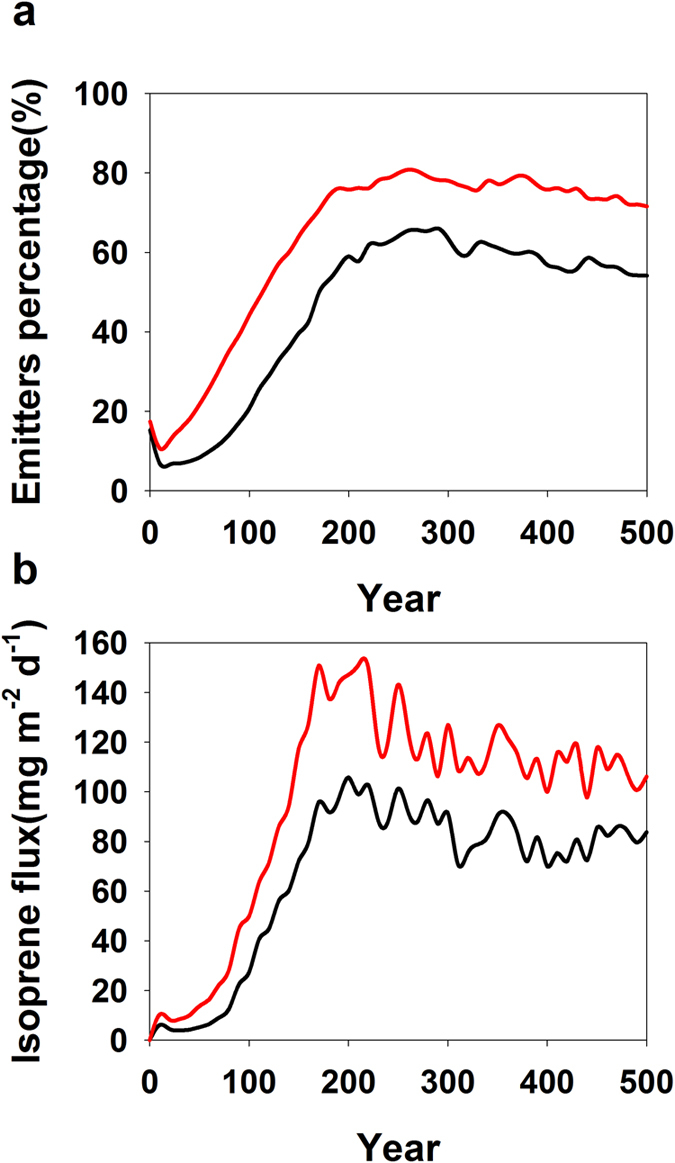
Successional changes in biomass of isoprene-emitting species and isoprene flux. The percentage of total biomass comprised by isoprene-emitting species (10 species in total) (**a**). Dynamics of average daily isoprene flux over the July of each year during succession (**b**). Dark and red line denote without and with O_3_ stress, respectively.

We examined the species composition change in terms of isoprene-emitting species. Because isoprene-emitting species tend to be better protected against atmospheric oxidative pressure (*e.g.,* refs 24, 25, 26), the proportion of isoprene-emitting species in the simulated forest increases significantly from 60% to 80% under O_3_ stress (Fig. 3a). Among the emitting species, ‘other’ species represents a high percentage (~50%) at the beginning of the succession (Supplementary Fig. 6). However, these species are almost completely replaced at about 60 years by four isoprene-emitting oak species (*Q. alba, Q. velutina, Q. rubra*, and *Q. prinus*). From these simulations, tropospheric O_3_ pollution modifies forest composition and favors isoprene-emitting species. At the same time, tropospheric O_3_ pollution engenders a decline of forest biodiversity as proposed earlier^23^.

We simulated the isoprene emission from this forest to investigate these implied feedbacks. Isoprene flux increases sharply within the first 200 years of compositional dynamics, and remains relatively stable with a slightly decline over the remaining simulation with some inter-annual variability (Fig. 3b). Emitters are often shaded by non-emitting species (*e.g., L. tulipifera*) early in succession and are then more exposed to light when they eventually become canopy dominants, which is indicated by, for example, the change of sunlit leaf area proportion and light extinction for a *Q. alba* tree’s canopy at 10 and 300 years (Supplementary Fig. 4). We also calculated the dynamics of sunlit versus shaded leaf area index (LAI), and the corresponding isoprene flux initially increases and then stabilizes (Supplementary Fig. 5). The sunlit LAI is small relative to shaded LAI, but the sunlit leaf-derived flux always dominates in its contribution to the total isoprene flux. It accounts for ~70% of the isoprene flux in the later successional forest (Supplementary Fig. 5). The contribution to emission from early successional species is initially large but declines quickly (Supplementary Fig. 6). As succession progresses, the isoprene flux becomes dominated by the aforementioned four oaks (*Q. alba, Q. velutina, Q. rubra,* and *Q. prinus*). Dynamic change in forest composition significantly alters the simulated isoprene flux under elevated O_3_ conditions (Fig. 3b). On average, the isoprene flux is increased by 50% (from 80 mg m^−2^ d^−1^ to 120 mg m^−2^ d^−1^) under O_3_ stress.

## Discussion

These simulations suggest that O_3_ pollution does not necessarily cause reduced forest productivity or carbon storage. The FACE study by Zak *et al.*^13^, which included both O_3_-tolerant and –sensitive species or genotypes and reported unsuppressed net primary productivity after long-term fumigation, supports this conclusion. In contrast, the earlier modeling studies that have found such reductions^1^,^2^,^3^ have explicitly not included species-specific effects and thus have not produced these compensatory responses. One would expect agricultural systems, which lack the interspecific dynamics and plant-size differences simulated here, to feature the O_3_-generated productivity reductions^5^.

Previous comparative work on managed and unmanaged systems has measured the effects of forest composition on isoprene emissions^27^, and the results described here are congruent. In forests, enhanced isoprene emission arising from species-composition changes represents a potential positive feedback loop. If O_3_ tolerance is linked to isoprene production, as has been suggested (*e.g.,* refs 24, 25, 26), these simulations of temperate deciduous forest in southeastern USA can be extended to other types of forests (tropical and boreal forests) with global-scale implications.

Three important implications emerge from this study. The first is that community dynamics, in particular compensatory responses and competitive release, suggest that O_3_ may not play a substantial role in depressing productivity and carbon storage at ecosystem and landscape scales. Second, many other large-scale environmental perturbations that are occurring today also have species-dependent effects, *e.g.,* rising CO_2_ concentrations, increasing temperatures, and nitrogen deposition^28^,^29^,^30^,^31^. For large-scale environmental perturbations that modify interactions among individual plants, changes in competitive relations can induce compensatory (or, potentially, synergistic) responses not inferred from aggregated models. Moreover, how these factors act together to affect the terrestrial ecosystems are far more important. Third, the ozone-diversity-isoprene emission feedback suggests connectivity between species-specific metabolism and atmospheric chemistry. This has only rarely been demonstrated^32^, but it implies the possibilities for a diverse array of interactions between the biosphere and the atmosphere. Future ecological and biosphere-atmosphere research should examine explicitly, rather than ignore by design, the potential for such species-specific impacts.

## Methods

### Description of UVAFME

UVAFME (Supplementary Fig. 1) simulates the growth, death, and regeneration of each individual tree annually on a 1/20 ha plot. Its dynamics are constrained by temperature, light, soil moisture, soil nutrient, wind, and fire conditions. Competition among trees for light, nutrient, and water resources are also included. The community dynamics and composition, including tree number of each species, basal area, leaf area, litter carbon and nitrogen, and biomass carbon and nitrogen, can be determined from processing the sizes and species of individual trees, which are computed annually in the model. The soil carbon, nitrogen, and water dynamics, along with soil carbon and nitrogen storage, soil respiration, and evapotranspiration, are calculated as state variables. These parameters include species-related parameters (quantifying species’ fundamental silvics and responses to environmental factors) and site conditions (*i.e*., local soil physiochemical properties and meteorological temperature and precipitation). More details concerning the model algorithms are referred to refs 18,33.

### Coupling with isoprene emission model

The canopy of each tree of an isoprene-emitting species is divided into 5 layers. Hourly isoprene emissions from sunlit and shaded leaves of each layer are determined by leaf area and standard emission rate, and constrained by hourly air temperature and leaf-level PPFD (photosynthetic photon flux density). The sunlit-leaves flux and the shaded-leaves flux sum to the hourly flux, which can be added together to obtain the daily flux (mg m^−2^ day^−1^) for each tree. The sum of isoprene emission of each tree is the canopy isoprene flux.

Emitting species and their standard emission rates are according to ref. 34 (Supplementary Table 1). Leaf area of UVAFME changes annually and we assume that the leaf area during July is constant. The leaf area is assumed to be uniformly distributed for each tree in the UVAFME.

Temperature-dependency algorithm of isoprene emission^35^ is:


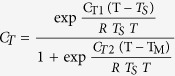
where R = 8.314 J K^−1^ mol^−1^, 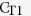
= 95,000 J mol^−1^, 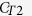
= 230,000 J mol^−1^, 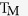
= 314 K, and 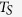
= 303 K. T is leaf temperature, which is assumed to be equal to hourly air temperature and through the canopy. Hourly temperature is calculated from daily minimum and maximum temperature, the previous-day maximum temperature, and the following-day minimum temperature (see Supplementary Note)^36^.

Light-dependency algorithm^35^ is:


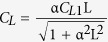
where L is leaf level PPFD (μmol m^−2^ s^−1^), α = 0.0027, and 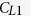
= 1.066. The hourly leaf-level PPFD at each canopy layer for sunlit and shaded leaves (the distribution of sunlit leaf area within a canopy can be described by an exponential model analogous to Beer’s law with the extinction coefficient for direct beam but without the light intensity multiplier) of each isoprene-emitting tree is achieved by three steps of calculations: First, above-forest stand PPFD is obtained; second, considering the shading by taller and surrounding trees, the light intensity above each isoprene-emitting tree within the forest stand is then calculated; and, third, the sunlit-leaf area, shaded-leaf area, and the corresponding PPFD on sunlit and shaded leaves at each canopy layer for each isoprene-emitting tree are calculated. In detail, direct beam and diffuse PPFD above the forest stand are calculated from incoming extraterrestrial solar radiation using an atmospheric transmissivity value of 0.6. Light intensity at each canopy layer within the canopy are determined by Beer’s law with different extinction coefficients for direct beam and diffuse light based on an assumption of spherical leaf angle distribution with accounting for light reflection and scattering. Light intensity on a shaded leaf is from both incoming diffuse light and scattered light from the direct beam. For more details concerning calculation of the sunlit and shaded leaf area and PPFD level, please refer to MEGAN 2.1 (ref. 21) and ref. 36.

### Input parameters estimation

Thirty-two species native to the southern Appalachian region in USA, including both deciduous and coniferous trees, are simulated. Twenty-four parameters required as inputs for each species were estimated (Supplementary Data 1). Specifically, wood bulk density values were from a global wood density data compiled by ref. 37. Species response to nutrient availability is according to ref. 38. All the remaining are estimated according to refs 39,40. Thirty years meteorological data of monthly precipitation (mm) and monthly maximum and minimum temperature (°C) ranging from 1981 to 2010 were obtained from a nearby NOAA (National Oceanic and Atmospheric Administration) meteorological station, Oak Ridge ATDD, Tennessee, USA (GHCND: USW00003841; Latitude/Longitude: 36.0028°/−84.2486°; Elevation: 275.8 m) to compute monthly average precipitation, monthly maximum and minimum temperature, and their standard deviations. Additionally, soil-related parameters including organic layer carbon and nitrogen, active layer carbon and nitrogen, and base soil carbon are estimated according to refs 41,42. Default values of 25 cm and 12.5 cm were used for soil field capacity and soil permanent wilting point, respectively.

### Modelling O_3_ effects on growth

To incorporate the O_3_ effects on tree growth into UVAFME, we first classify the 32 species into three categories based on their relative sensitivity to O_3_ stress: resistant, intermediate, and sensitive (Supplementary Table 1). This categorization derives from the current literatures including review studies^4^,^7^,^8^,^43^,^44^ and reports on individual species^45^,^46^,^47^,^48^,^49^,^50^,^51^. A growth reduction of 0, 10%, and 20% is exerted on resistant, intermediate and sensitive species, respectively (For a validity check of these specific reduction values, see Supplementary Fig. 7).

### Simulation methods

We apply a Monte Carlo simulation of a landscape of indeterminate size sampled with a system of independent sample plots with the same climate and soil conditions. Therefore, the average of the simulation corresponds to a shifting-mosaic steady-state landscape. An analysis of convergence of average species-specific biomass values finds that 150–200 replicate plots are necessary to provide a sample which approximates the landscape response of the forest^52^. Therefore, the model is run on a plot size of 500 m^2^ starting from bare ground and lasting for 500 years for 200 independent plots. All the results presented are the average of 200 such runs.

## Additional Information

**How to cite this article**: Wang, B. *et al.* Forests and ozone: productivity, carbon storage, and feedbacks. *Sci. Rep.* **6**, 22133; doi: 10.1038/srep22133 (2016).

## Supplementary Material

Supplementary Dataset

Supplementary Information
