# Supplementary material for: Forests and ozone: productivity, carbon storage, and feedbacks
Source: Sci Rep. 2016 Feb 22;6:22133. doi: 10.1038/srep22133 (PMC4762018; doi:10.1038/srep22133)
Supplement: Supplementary Information [file srep22133-s2.pdf]

Supplementary Information for

Forests and ozone: productivity, carbon storage, and feedbacks

Bin Wang, Herman H. Shugart, Jacquelyn K. Shuman & Manuel T. Lerdau*

Department of Environmental Sciences, University of Virginia, PO Box 400123, Clark Hall, 291 McCormick Road, Charlottesville, VA 22904-4123, USA.

*Correspondence to: mlerdau@virginia.edu (M.T.L.)

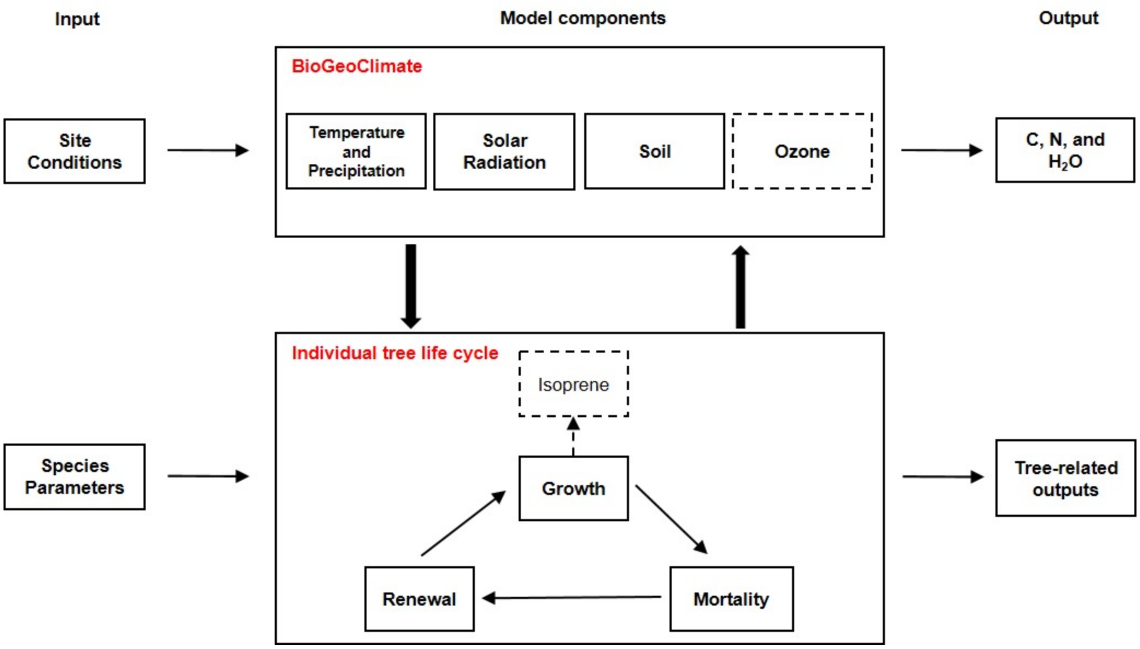

Supplementary Figure 1 Schematic of UVAFME structure and components. Dashed boxes denotes modifications made to simulate isoprene emission and to incorporate ozone effects.

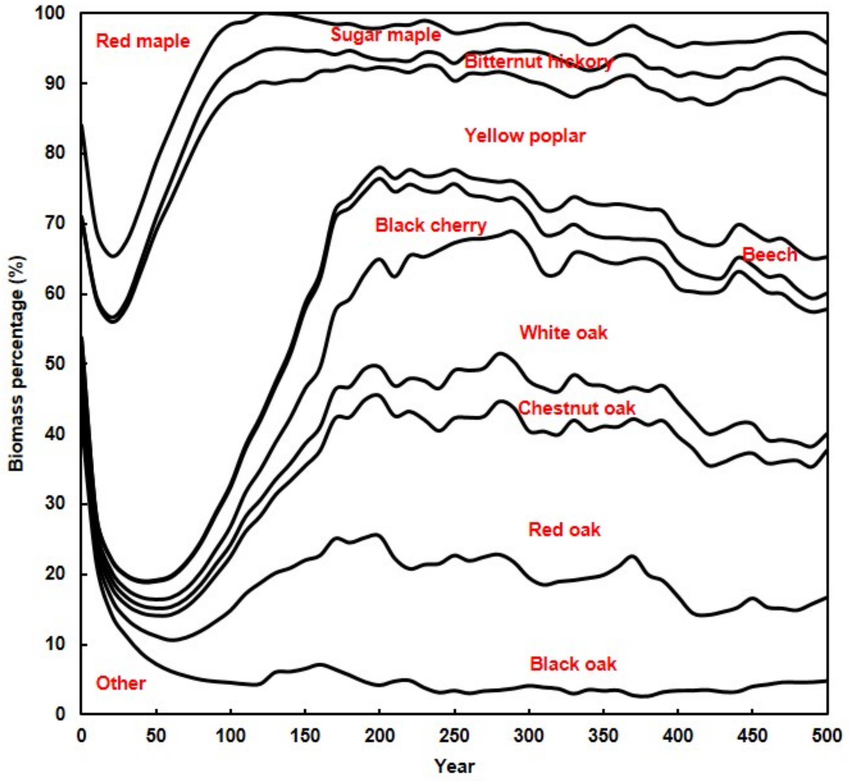

Supplementary Figure 2 Succession of the deciduous forest over 500 years without O_3. The vertical distance between two lines represents the percentage of total biomass comprised by each species. The 'other' species refers to all the remaining 22 species except for the 10 listed. Red maple- *Acer rubrum*, sugar maple-*Acer saccharum*, Bitternut hickory-*Carya cordiformis*, Yellow poplar-*Liriodendron tulipifera*, beech-*Fagus grandifolia*, white oak-*Quercus alba*, chestnut oak-*Quercus prinus*, red oak-*Quercus rubra*, and black oak-*Quercus velutina*.

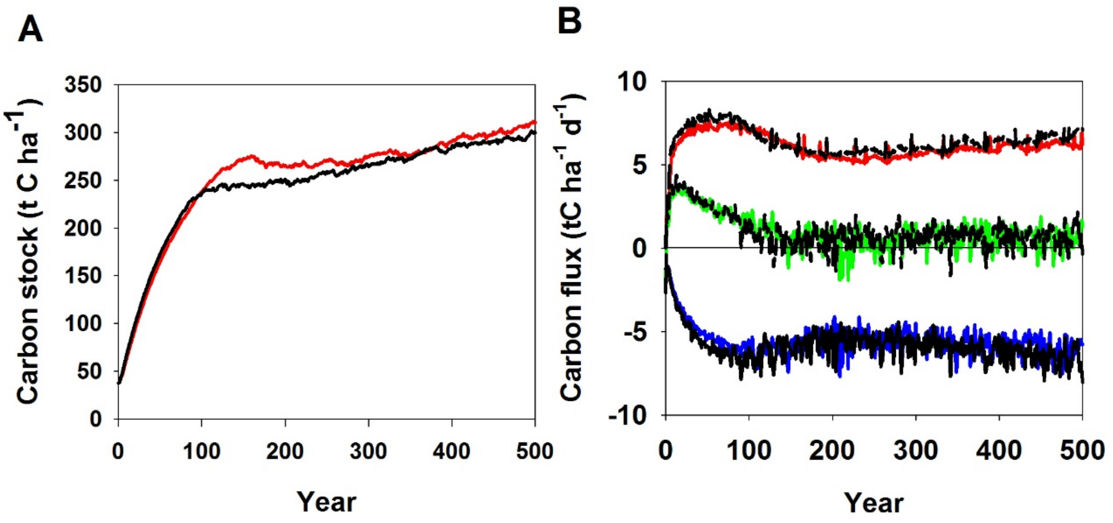

Supplementary Figure 3 Forest carbon stock and flux changes over succession due to O_3. Forest carbon (biomass and soil carbon) dynamics over succession (dark and red line denote without O_3 and with O_3, respectively) (**A**). Forest NPP (net primary productivity, red), soil respiration (blue), and NEP (net ecosystem productivity, green) responses to O_3 over the succession (**B**).

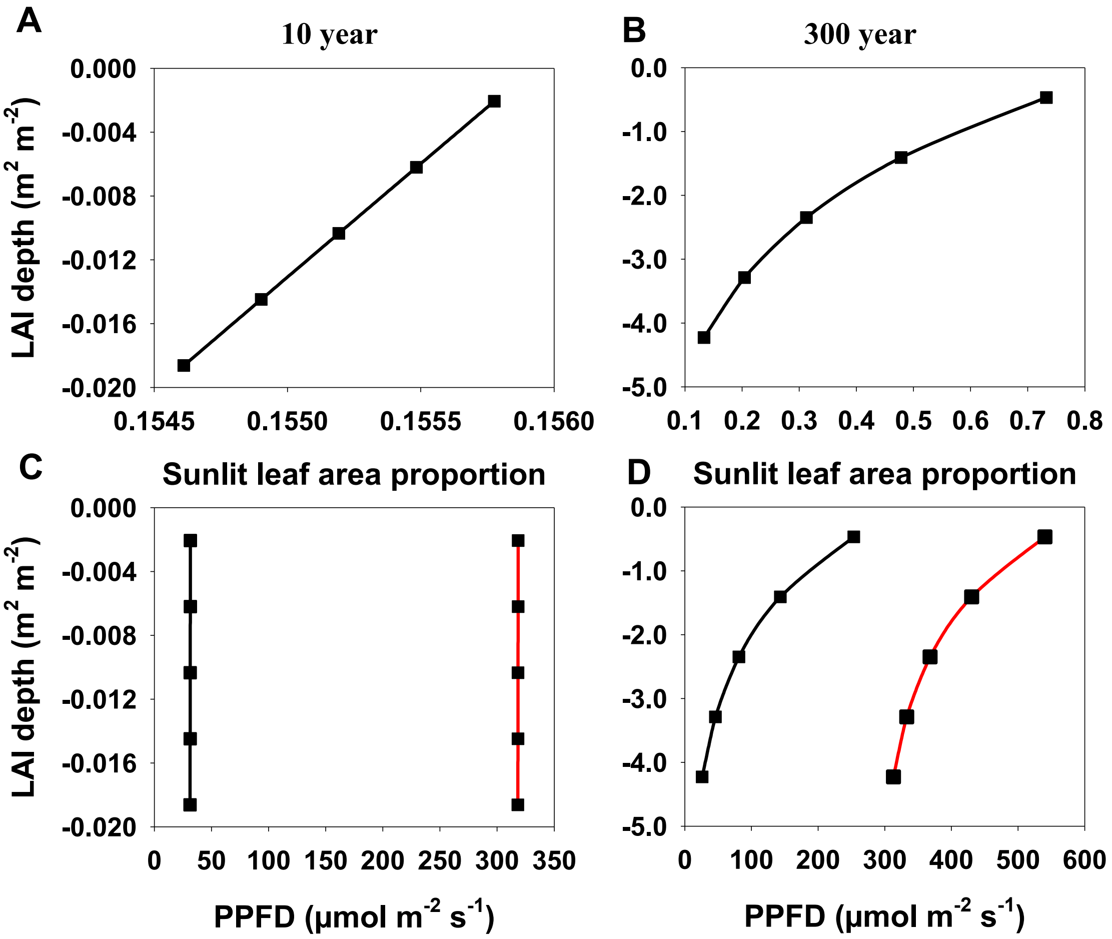

Supplementary Figure 4 Dynamics of sunlit leaf area fraction and sunlit and shaded leaf-level PPFD (photosynthetic photon flux density) profile. Sunlit leaf area fraction gradient (**A, B**) and sunlit leaf-level and shaded leaf-level PPFD changes (**C, D**) within the canopy of only one tree of *Quercus alba*, which is randomly chosen from the simulated forest stand at 10 year (**A, C**) and 300 year (**B, D**). Values represent the 13:00 of day 201 in each year. The negative axis values mean increased canopy depth. The change in light levels shown by the two trees of the same species at two different stages of the forest stand development demonstrate that because of forest composition and

structure change the shade-tolerant species (here *Quercus alba*) will become dominant in the forest, enabling leaves of this species to receive more light.

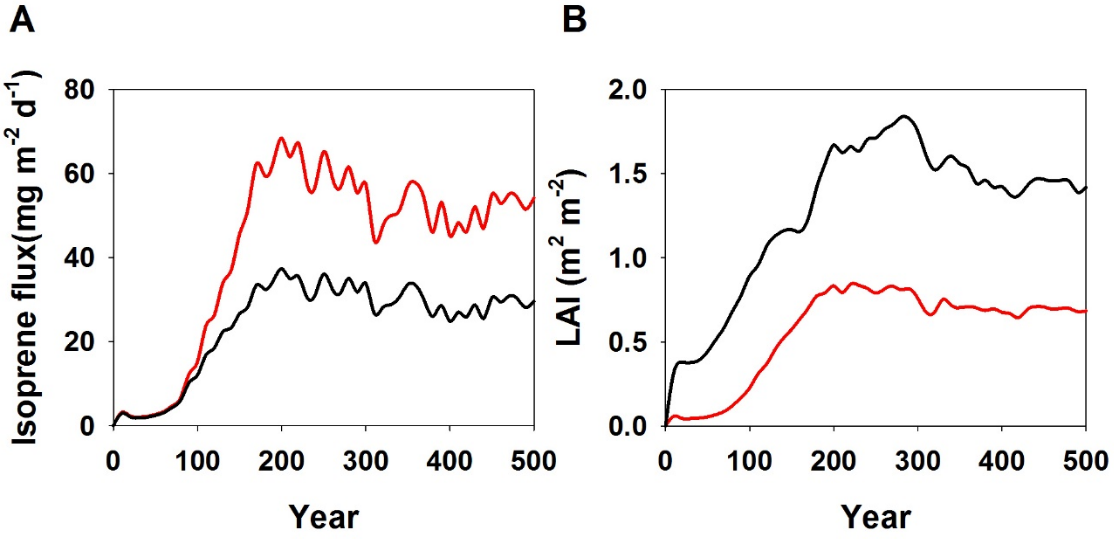

Supplementary Figure 5 Dynamics of isoprene emission from sunlit and shaded leaves over succession. Isoprene flux is calculated from both sunlit (red) and shaded leaves (black). The isoprene emission (**A**) and LAI (leaf area index; **B**) represent the values at 13:00 of day 201 from each year.

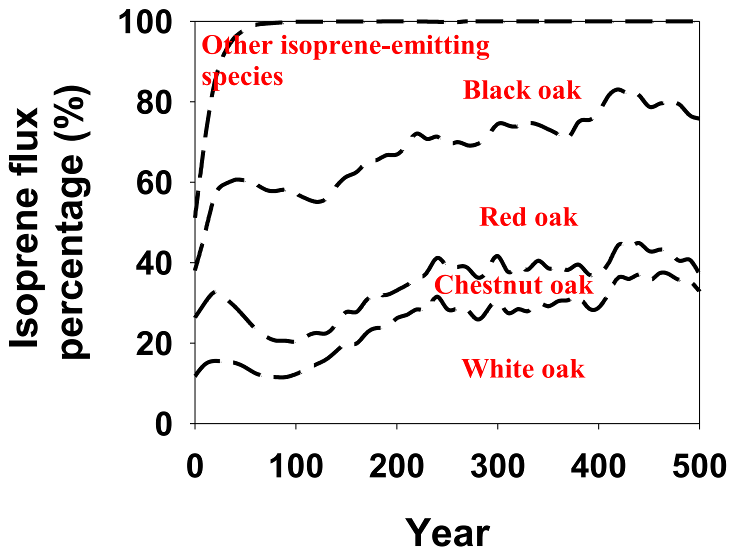

Supplementary Figure 6: Relative contribution to total isoprene emissions by individual species without O_3 pressure. The interval between two lines represents the percentage of total isoprene emissions comprised by, from upper to bottom, other isoprene-emitting species, black oak, red oak, chestnut oak, and white oak, respectively. Note the biomass change of these isoprene-emitting species are basically same with their isoprene emission dynamics. Hence, the graph is not shown.

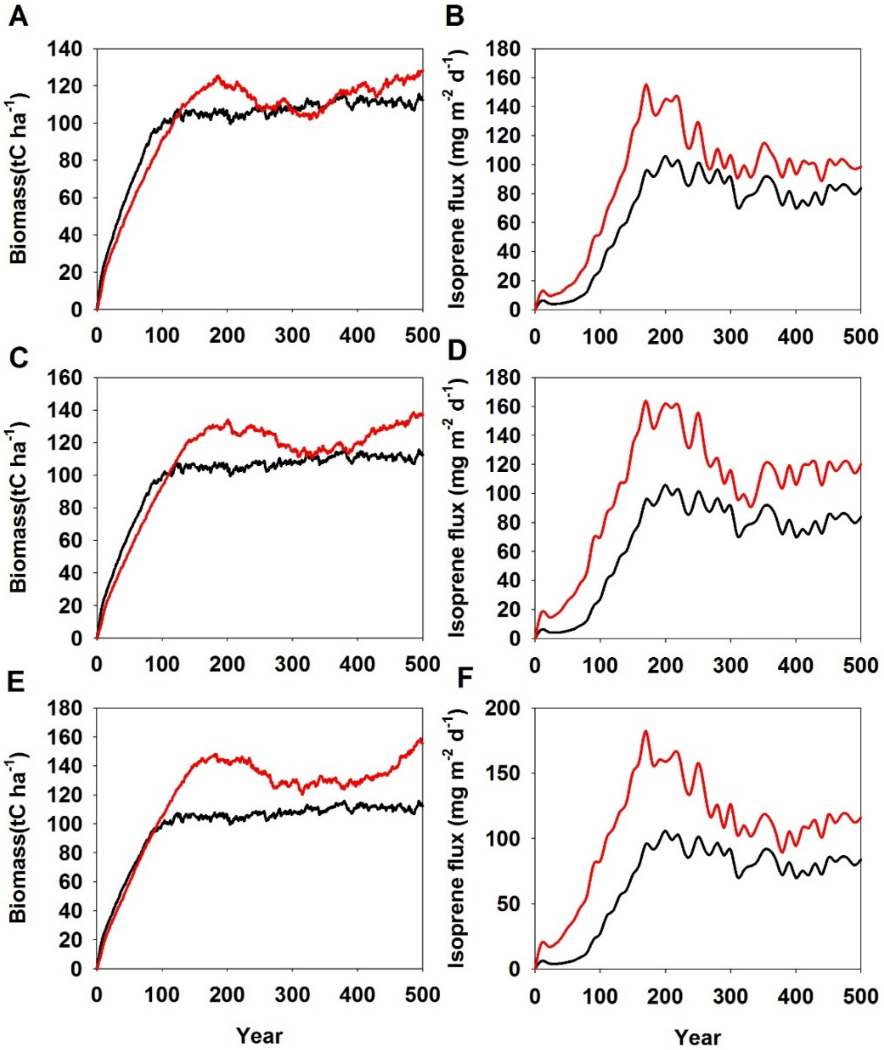

Supplementary Figure S7 Responses of biomass and isoprene flux to O_3 stress of different levels. A growth reduction of 10%, 20%, and 30% for resistant, intermediate, and sensitive species, respectively (**A, B**); of 10%, 20%, and 40%, respectively (**C, D**); and of 0, 10%, and 40%, respectively (**E, F**) are applied to drive the simulations. Such various combinations of ozone-induced growth reduction for species of different sensitivity always show unsuppressed biomass accumulation and enhanced isoprene

flux. Hence, these results are presented to argue that the modelling results discussed throughout this work with a growth reduction of 0, 10%, and 20% for resistant, intermediate, and sensitive species, respectively, are convincing.

Supplementary Table S1 Species-specific sensitivity to O_3 and standard isoprene emission rate.

Resistant	Rate(nmol m^{-2} s^{-1})	Intermediate	Rate(nmol m^{-2} s^{-1})	Sensitive
Carya cordiformis		*Acer saccharum*		*Acer rubrum*
Carya glabra		*Cornus florida*		*Aesculus octandra*
Carya ovata		*Diospyros virginiana*		*Cercis canadensis*
Carya tomentosa		*Juglans nigra*		*Fraxinus americana*
Fagus grandifolia		*Juniperus virginiana*		*Liriodendron tulipifera*
Nyssa sylvatica	30	*Liquidambar styraciflua*	35	*Oxydendron arboreum*
Quercus alba	50	*Quercus coccinea*	50	*Prunus serotina*
Quercus falcata	57	*Quercus velutina*	72	*Pinus echinata*
Quercus prinus	23	*Robinia pseudoacacia*	45	*Pinus strobus*
Quercus rubra	30	*Tilia heterophylla*		*Pinus virginiana*
Quercus stellata	50			*Sassafras albidum*

Supplementary Note

The following Fortran code subroutine represents the detailed procedures used to calculate the hourly temperature from daily minimum (tmin) and maximum temperature (tmax), the previous-day maximum temperature (tmaxb), and the following-day minimum temperature (tmina). The references used to develop the equations represented in this code are all given in the main text:

```
Subroutine hourlytemperature(tmaxb,tmin,tmax,tmina,dayl,hour,hourtemp)


     real,                          intent(in)  :: tmaxb,tmin,tmax,tmina
     integer,                       intent(in)  :: hour
     real,                          intent(in)  :: dayl
     real,                         intent(out)  :: hourtemp
     !local variables
     real, parameter   :: pi = 3.14159,tc = 4., p = 1.5
     real              :: tsunst,nightl,hourtemp1,sunris,sunset

     sunris = 12. - 0.5*dayl
     sunset = 12. + 0.5*dayl

     if (hour .lt. sunris) THEN
      tsunst = tmin +(tmaxb - tmin)*sin(pi*(dayl/(dayl+2.*p)))
      nightl = 24. - dayl
      hourtemp1 = (tmin-tsunst*exp(-nightl/tc) +    &
                 (tsunst - tmin)*exp(-(hour + 24. - sunset)/tc))/  &
                 (1. - exp(-nightl/tc))

     else if(hour .lt. 13.5) then
       hourtemp1 = tmin + (tmax -tmin)*sin(pi*(hour-sunris)/(dayl + 2.*p))

     else if(hour .lt.sunset) then
       hourtemp1 = tmina +(tmax-tmina)*sin(pi*(hour-sunris)/(dayl + 2.*p))

     else

       tsunst = tmina + (tmax-tmina)*sin(pi*(dayl/(dayl+2.*p)))
       nightl = 24. - dayl
       hourtemp1 = (tmina-tsunst*exp(-nightl/tc) +                    &
                  (tsunst - tmina)*exp(-(hour-sunset)/tc))/          &
                  (1.- exp(-nightl/tc))
     end if

     hourtemp = hourtemp1 + 273.15
     return
```

```
end subroutine
```
